# Nonrandom dispersal drives phenotypic divergence within a bird population

**DOI:** 10.1002/ece3.563

**Published:** 2013-11-06

**Authors:** Carlos Camacho, David Canal, Jaime Potti

**Affiliations:** Department of Evolutionary Ecology, Estación Biológica de Doñana—CSICAv. Américo Vespucio s/n, 41092, Seville, Spain

**Keywords:** Dispersal, *Ficedula hypoleuca*, habitat preferences, phenotypic divergence, pied flycatcher, population structure

## Abstract

Gene flow through dispersal has traditionally been thought to function as a force opposing evolutionary differentiation. However, directional gene flow may actually reinforce divergence of populations in close proximity. This study documents the phenotypic differentiation over more than two decades in body size (tarsus length) at a very short spatial scale (1.1 km) within a population of pied flycatchers *Ficedula hypoleuca* inhabiting deciduous and coniferous habitats. Unlike females, males breeding in the deciduous forest were consistently larger than those from the managed coniferous forest. This assortment by size is likely explained by preset habitat preferences leading to dominance of the largest males and exclusion of the smallest ones toward the nonpreferred coniferous forest coupled with directional dispersal. Movements of males between forests were nonrandom with respect to body size and flow rate, which might function to maintain the phenotypic variation in this heritable trait at such a small spatial scale. However, a deeply rooted preference for the deciduous habitat might not be in line with its quality due to the increased levels of breeding density of hole-nesting competitors therein. These results illustrate how eco-evolutionary scenarios can develop under directional gene flow over surprisingly small spatial scales. Our findings come on top of recent studies concerning new ways in which dispersal and gene flow can influence microevolution.

## Introduction

Understanding the causes of population divergence is a central issue in evolutionary biology (Schluter 2000). Many studies have suggested that evolutionary divergence of natural populations often reflects the balance between diversifying natural selection and homogenizing gene flow (Slatkin 1987; Garcia-Ramos and Kirkpatrick 1997; Lenormand 2002; Hendry and Taylor 2004). However, although the crucial role of natural selection in adaptive divergence has been empirically confirmed (Endler 1986; Schluter 2000), the extent to which gene flow constrains evolution remains controversial (Blondel et al. 1999; Lenormand 2002; Senar et al. 2002, 2006; Hendry and Taylor 2004; Garant et al. 2005, 2007; Postma and van Noordwijk 2005; Porlier et al. 2009, 2012). Recently, a growing number of studies is changing the predominant view on the antagonistic role of natural selection and gene flow in shaping population divergence, revealing new ways in which gene flow can drive microevolution (e.g., Garant et al. 2005; Postma and van Noordwijk 2005; Senar et al. 2006). For instance, nonrandom movements with respect to phenotype may influence population divergence and evolution on ecological time scales (Duckworth 2006; Price 2008; Bolnick et al. 2009; Clobert et al. 2009; Ravigné et al. 2009; reviewed by Edelaar and Bolnick 2012).

In the context of habitat selection, theory predicts a positive relationship between the dispersers' phenotype and the quality of postdispersal, settlement habitats (Stamps 2006; Edelaar et al. 2008; Bolnick et al. 2009). In fact, the few studies that have explored how intraspecific phenotypic variation relates to dispersal and settlement patterns in group-living (Seddon et al. 2004) and nonsocial (Garant et al. 2005; Senar et al. 2006) songbirds have found support for this prediction. However, the opposite pattern may also occur due to, for example, perceptual traps leading to mismatches between phenotype and habitat quality (see Patten and Kelly 2010). Finding out the extent to which this somewhat counterintuitive process occurs in the wild may help to fill an important gap in our understanding of how ecological processes can lead to contemporary evolutionary scenarios.

Processes influencing population viability (e.g., local recruitment and evolutionary response to natural selection) rarely act in isolation, but may interact with each other through the dispersal decisions of individuals (Benard and McCauley 2008). Deeply rooted habitat preferences and imprinting on natal habitats may create unidirectional gene flow and thus asymmetries in the geographic patterns of local adaptation and maladaptation (Kawecki and Holt 2002; Davis and Stamps 2004; Vallin and Qvarnström 2011). In fact, habitat selection could be maladaptive itself, especially for migratory birds, as habitat preference may not always match habitat quality (Hollander et al. 2011). After arrival from wintering quarters, migrant birds are often time constrained when choosing their breeding location (Newton 2008). As a result, the slightest setback may force individuals to settle in suboptimal habitats instead of further exploring adjacent environments for acquiring better quality territories (Battin 2004; Hollander et al. 2011). Moreover, under hierarchy regimes governing spatial distribution, larger, usually dominant individuals, should be found disproportionately often within the preferred habitat type (Duckworth 2006; Robertson and Hutto 2006). Across time, directional dispersal may also create asymmetries in breeding density and thus potentially influence habitat quality (Battin 2004; Garant et al. 2005), while changes induced by humans may moderate those differences and/or lead to crucial resources for birds suddenly arising in surrounding environments (Robertson and Hutto 2006). As a consequence, individuals that otherwise might have been unable to acquire breeding territories in preferred habitats may have an opportunity to settle in human-transformed environments.

The pied flycatcher (*Ficedula hypoleuca*) is a long-distance migrant passerine which prefers deciduous over coniferous forests for breeding (Alatalo et al. 1985; Lundberg and Alatalo 1992; Sanz 1995). As a result, after arrival from wintering areas, preference for certain habitats should lead to competitive interactions before definitive settlement to breed (see Lundberg et al. 1981). Male pied flycatchers arrive before females, search for a suitable tree hole or nest box to breed, and compete, sometimes fiercely, for its possession (Alatalo et al. 1994). Size is important in male–male interactions, with larger individuals usually winning territorial contests (Alatalo et al. 1985; Sirkïa and Laaksonen 2009) while females choose among those males already owning a suitable cavity (Dale and Slagsvold 1990; Lundberg and Alatalo 1992). Accordingly, we would expect that, while no clear pattern related to body size should appear in females, smaller subdominant males, unable to win competitive interactions with larger individuals for highly regarded territories in deciduous patches would be relegated to the underappreciated coniferous forest. Here, we explore this possibility using a long-term data set from a population of pied flycatchers inhabiting contrasting nearby environments. We also test whether dispersal asymmetries may arise from the interplay between an individual's propensity (behavior) to disperse and its phenotype-dependent ability to succeed in settling.

## Methods

### Study system and general procedures

**Figure 1:**
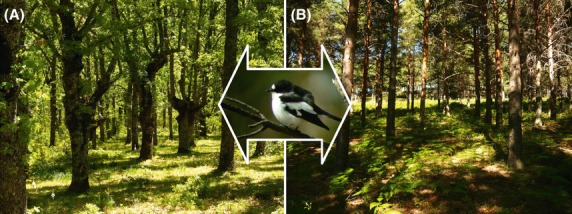
Study system, showing a male pied flycatcher *(Ficedula hypoleuca)* and the two study habitats; (A) *Quercus pyrenaica* oakwood, (B) *Pinus sylvestris* plantation. Photos by Carlos Camacho.

Data were obtained from a population of pied flycatchers in central Spain (ca. 41°N, 3°W, 1200–1300 m asl) during a long-term study conducted from 1988 to 2011 in two different montane habitats: an old deciduous oak (*Quercus pyrenaica*) forest (DF) and a managed mixed coniferous (mainly *Pinus sylvestris*) forest (CF) separated by a minimum distance of 1.1 km (Fig. 1). Sampling intensity was limited in 2002–2003, these years are therefore excluded from analyses. At the beginning of the study, pied flycatchers were confined to natural tree holes in DF (Carrascal et al. 1987; Potti and Montalvo 1990), and no cases of breeding had been observed in CF due to lack of natural cavities. In 1984 (DF) and 1988 (CF), wooden nest boxes (172 and 81, respectively) at a mean distance of 30 m (SE 14.1) were provided and have been maintained until nowadays. Because only one nest was occupied in CF in 1988, that year was also excluded from the comparisons with DF. Nest boxes are also used by tits (Paridae), treecreepers (*Certhia brachydactyla*), and nuthatches (*Sitta europaea*) in both forests.

Nest boxes were regularly checked to ascertain breeding phenology. Adult breeding birds were captured while incubating (females) or feeding nestlings (both sexes) by means of a nest box trap. Birds were individually marked with color and metal rings, measured for tarsus length (±0.05 mm), and aged as either yearling or older following criteria in Karlsson et al. (1986) and Potti and Montalvo (1991a). Unringed birds aged as after their second calendar year when first captured were assigned an age of 2 years on the basis of patterns of age at first breeding in birds of exactly known age (Potti and Montalvo 1991b). All fledglings were marked with metal rings at the age of 13 days old.

### Estimates of breeding density

Temporal changes in breeding densities of pied flycatchers were followed to assess the possible effects of intraspecific interactions on population dynamics. Overall breeding density was determined by quantifying yearly nest box occupancies either by flycatchers or other species (see, e.g., Blondel et al. 1999; Garant et al. 2004), whereas its reciprocal (i.e., empty nest boxes) was used as a relative measure of territory (nest site; von Haartman 1956) availability. The proportion of nest boxes occupied by other hole-nesting species in relation to those taken by pied flycatchers (see Canal et al. 2012) was used as a proxy for the relative intensity of interspecific competition within the guild of sympatric insectivorous birds with similar breeding requirements (Ulfstrand 1977).

### Habitat preferences and movements of individuals

Fidelity of birds to their birth or breeding habitats was used to assess the flow of individuals between both forests. The proportion of individuals breeding for the first time in their natal areas was used as a measure of philopatry, whereas adult site tenacity was assessed by the proportion of individuals breeding in the same area in the following years. Unmarked birds that were first caught as breeding adults were defined as being immigrants (Garant et al. 2004). Between-habitat differences in the annual rates of immigration, expressed as the number of new immigrants (males and females) in relation to the total number of breeding individuals, were also determined.

We assessed differences in tarsus length (a proxy for body size in songbirds; Senar and Pascual 1997) between natal dispersers and their potential competitors (see below) in each forest to explore the possible role of this trait in causing phenotypic divergence between habitats. As commonly reported in other migrants (Newton 2008), older, site-tenacious individuals breed consistently earlier (as scored by the social female's laying date of the first egg) than individuals breeding for first time (*F*_1,2216_ = 65.61, *P* < 0.001; see Potti 1998; Canal et al. 2012). Therefore, when building the pool of individuals to be compared with dispersers for nest sites, we assumed that, despite all males are possibly involved in competitive interactions with putative dispersers, only those breeding for the first time (either immigrant or native [i.e., hatched in our nest boxes]) are potential dispersers. To account for between-year variation in each habitat, tarsus measurements were standardized by subtracting the mean sex-specific annual values in each habitat from each individual measurement. Thus, two residuals for each individual exchanging habitats in its first breeding attempt were calculated for comparisons of its size relative to birds settled in both their natal and destination habitats.

Due to logistical reasons, choice experiments to estimate bird habitat preferences are often unfeasible in the field (Hollander et al. 2011). Therefore, we used individual size distribution, differences in site fidelity, and temporal variation in population size as surrogate measures of habitat preferences of pied flycatchers (Robertson and Hutto 2006).

### Statistical methods

To investigate the phenotypic differentiation in body size of males and females (both native and immigrant) between both habitats over years, we fitted Generalized Linear Mixed Models (GLMM with normal distribution and identity link functions). The models included tarsus length as the response variable and habitat type, year, and their interaction as fixed factors. Individual identity was included as a random effect to avoid pseudoreplication. To explore differences in size between potential dispersers, we ran a set of General Linear Models (GLM) including habitat type as a fixed effect. For these analyses, measurements of tarsus length were averaged when individuals were recaptured ≥2 years to minimize measurement error and avoid pseudoreplication. To analyze whether differences in breeders' densities (both pied flycatchers and other hole-nesting passerines) were stable over years, we fitted GLMs including year as a continuous variable, habitat type, and their interaction. The Wilcoxon signed-ranks test was used to analyze differences in annual rates of immigration between both habitats. Statistical analyses were made with SAS v. 9.2 (SAS Institute Inc 2008).

## Results

### Phenotypic divergence

Analyses of body size involved 1168 males and 1264 females (2045 and 2394 measurements of tarsus length, respectively). The across-years repeatability (Lessells and Boag 1987) of tarsus length is highly significant in both sexes (males: *R* = 0.78, females: 0.75, both *P* < 0.0001). Pairs formed randomly with respect to size in either habitat (both *r* < 0.03, *P* > 0.1).

**Figure 2:**
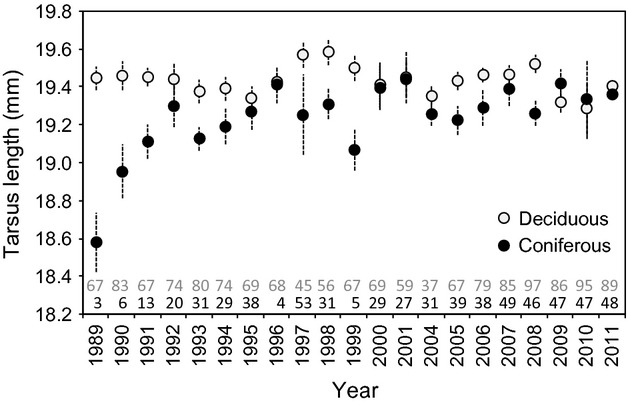
Annual variation in mean (±SE) tarsus length of adult male pied flycatchers in CF (black line, filled circles) and DF (gray line, open circles). The year 1988 is omitted because the only one male breeding in CF was not measured. Numbers above the *x*-axis denote sample sizes of males in CF (lower row) and DF (upper row).

Males breeding in CF were smaller than those from DF (mean ± SE: 19.31 ± 0.03 vs. 19.41 ± 0.02; GLMM: habitat: *F*_1,1_ = 13.49, *P* = 0.0003; year: *F*_1,23_ = 4.62, *P* < 0.0001), with a significant interaction (habitat × year: *F*_1,20_ = 2.66, *P* = 0.0001; Fig. 2) due to the opposite trend prevailing in only 3 of 21 years. On the contrary, females were of similar size in both habitats (mean ± SE: 19.42 ± 0.03 and 19.48 ± 0.02 in CF and DF, respectively; habitat: *F*_1,1_ = 3.15, *P* = 0.08; year: *F*_1,22_ = 2.91, *P* < 0.0001, after excluding the nonsignificant interaction term).

### Breeding density

**Figure 3:**
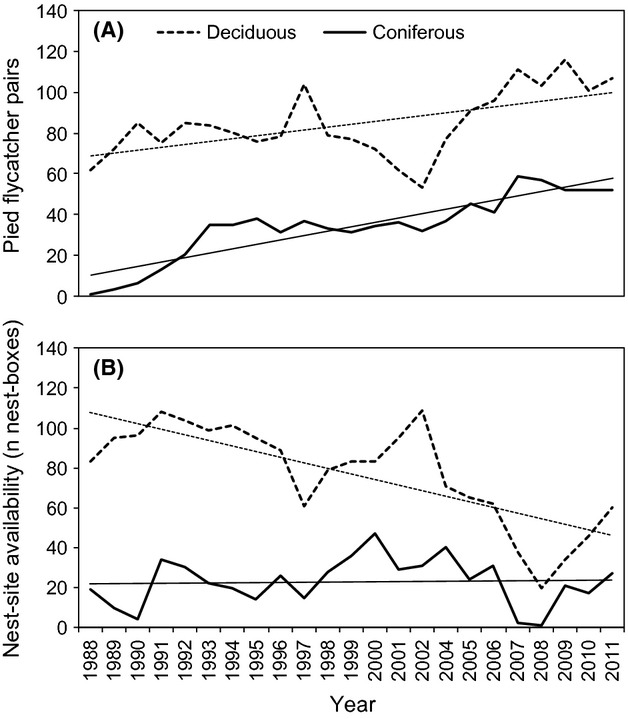
(A) Numbers of breeding pairs of pied flycatchers in CF and DF along the study period. (B) Nest-site availability, as measured by the number of nest boxes unoccupied either by flycatchers or other hole-nesting songbirds. Nest-site availability is inversely related to the overall density experienced by breeding birds.

The numbers of pied flycatchers increased similarly in both forests after providing them with an excess of nest boxes (GLM: year × habitat*: F*_1,42_ = 1.97, *P =* 0.17; Fig. 3A). In contrast, as nest box occupancy by competitors of pied flycatchers remained almost invariable over time in CF, but gradually increased in DF, the availability of nest sites differed significantly between habitats (GLM: year × habitat*: F*_1,42_ = 20.57, *P <* 0.0001), translating into differential overall breeding density (Fig. 3B). Thus, a simultaneous increase of pied flycatchers and competing hole-nesting species caused differences between both habitats in overall density of hole-nesting songbirds. It is worth noting that nest box occupation rate is higher in CF than DF due to the almost total absence of natural cavities in the former habitat.

### Movement and size of individuals exchanging habitats

**Figure 4:**
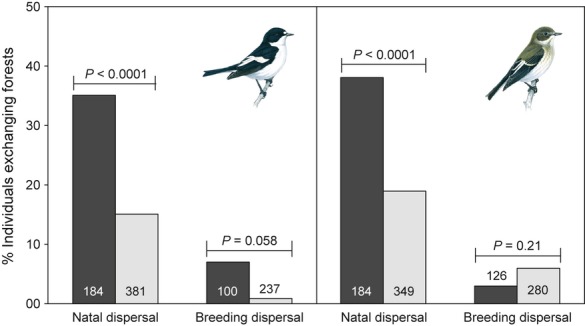
Natal and breeding dispersal of male (left) and female pied flycatchers between the coniferous (CF, dark bars) and the deciduous plots (DF, light bars). Figures within/beside bars are total numbers of individuals.

Natal dispersal was nonrandom with respect to the habitat of origin. The proportions of novice males and females leaving their coniferous natal habitat to breed in the adjacent deciduous habitat significantly exceeded those moving the other way round (Fig. 4). Although marginally nonsignificant, most of the (few) adult males that exchanged habitats also moved from CF to DF rather than the reverse (Fig. 4). Consequently, as expected from pied flycatcher habitat preferences, the proportion of birds that returned to their natal area was higher in DF than in CF, suggesting that philopatry is widespread among birds from the deciduous habitat. In contrast, after first breeding, most surviving breeding males and females from either habitat remained in their initial breeding habitat in the following years (Fig. 4).

**Figure 5:**
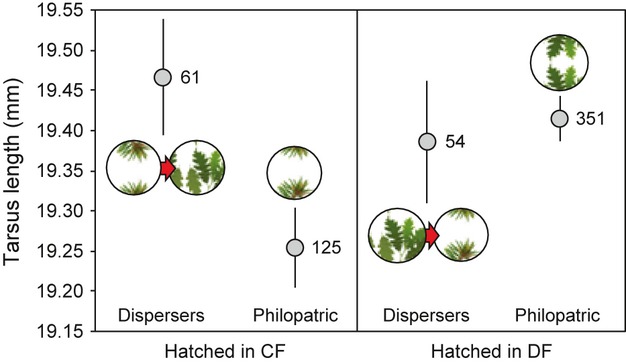
Variation in mean (±SE) tarsus length of male pied flycatchers in relation to dispersal behavior. Numbers beside bars are sample sizes.

Natal dispersal was furthermore size biased (Fig. 5), as novice males from CF that moved to DF were significantly larger than those that settled in their natal area (i.e., native males from CF, immigrants to CF, and dispersers from DF; GLM: *F*_1,386_ = 5.62, *P* = 0.018) and similar to those occupying their destination area (i.e., native males from DF and immigrants to DF; GLM: *F*_1,858_ = 0.15, *P* = 0.7). The few native birds that dispersed from DF to CF were of a size similar to that of novice males settled in DF (i.e., native males from DF, immigrants to DF, and dispersers from CF) or CF (i.e., native males from CF and immigrants to CF; GLM: *F*_1,912_ = 0.43, *P =* 0.51 and *F*_1,325_ = 1.33, *P =* 0.25, respectively). Thus, at the whole population level, males from the deciduous site (regardless of their dispersal behavior) are medium sized, whereas those from the coniferous site rank in both extremes of the body size range (Fig. 5). Both groups of dispersers (from DF or CF) did not differ in age (Mann–Whitney test, *U* = 914.5, *P* = 0.96), so a possible influence of age-related competitive ability may be discarded. In contrast to males, females dispersing to either habitat did not differ in size from those remaining in their natal or destination areas (all *P* > 0.35).

Immigrants to CF were larger than those to DF, although the reverse was true in 3 of the 20 years in which immigrant individuals were recorded (GLMM: habitat: *F*_1,1_ = 1.67, *P* = 0.2; year: *F*_1,22_ = 1.30, *P* = 0.17; habitat × year: *F*_1,19_ = 1.89, *P* = 0.01). Moreover, CF and DF experienced a roughly comparable and constant inflow of immigrant birds over time (Wilcoxon signed-ranks test, *T* = 133, *P* = 0.85).

## Discussion

We have shown that the intensity of dispersal and the phenotype of dispersers that finally settled to breed differed between habitats, driving phenotypic differentiation of pied flycatcher males in close proximity. That divergence was fueled by divergent patterns of natal dispersal in males, creating asymmetric exchanges between habitats in terms of body size. As a result, the largest males born in the coniferous forest moved to the deciduous forest while a small proportion of (mostly medium sized) males from the oakwood left their natal site to settle in the underappreciated, coniferous habitat.

Until providing nest boxes, pied flycatchers were confined to natural tree holes in the deciduous habitat (Potti and Montalvo 1990), as the lack of natural cavities prevented birds from breeding in the pinewood area. Once nest boxes were provided, flycatcher numbers gradually increased in both habitats (Potti and Montalvo 1990; Lundberg and Alatalo 1992). This is consistent with the results of Mänd et al. (2009), who suggested that provisioning with nest boxes enables raising breeding densities equally in both optimal and marginal habitats. However, with strong differences in the availability of food resources and/or of competitors exploiting them, long-term demographic consequences at the community level in contrasting habitats could evolve in opposite ways. In our study system, the temporal dynamics of the nest box breeding community differed between habitats as overall breeding density increased only within DF, whereas it remained relatively constant in CF.

Several previous studies have found density-dependent effects to be the ultimate cause of phenotypic divergence between songbird populations although density dependence is unlikely to be the cause of the differentiation in male size in our study system. Providing nesting sites in already attractive habitats could result in a supraoptimal breeding density, possibly leading to a density-driven ecological trap (Mänd et al. 2005, 2009). Density-related differences in habitat quality could also bias settlement decisions toward the low-density area and, ultimately, lead to the evolution of phenotypic divergence at small spatial scales (Senar et al. 2002; Garant et al. 2005; Porlier et al. 2012). Nevertheless, the reverse is true in our study system, where patterns of settlement are biased toward the high density, deciduous plot. Alternatively, decisions on where to settle could be the outcome from optimally biased spatial segregation, where individuals would be accurately matched to those habitats where their phenotypes would perform best (matching habitat choice, reviewed in Edelaar et al. 2008). Experiments testing for nonrandom gene flow or dispersal could throw light into a possible role of matching habitat processes in promoting phenotypic differentiation in pied flycatchers (Edelaar and Bolnick 2012) but, unfortunately, they are hard to implement in free-ranging vertebrate populations.

Long-term data on population densities and strong evidence for nonrandom dispersal suggest that settlement preferences of pied flycatchers have remained unchanged across more than the two decades covered by this study. The numbers of individuals of either sex born in CF that dispersed to settle in DF notably exceed those moving the other way round, whereas larger, dominant males native from either habitat preferred the deciduous forest, despite the higher population density levels therein. Our data further show that a phenotype–dispersal interaction underlies the size-based habitat distribution of pied flycatchers in which the largest and potentially dominant males from CF move to DF, whereas the smallest males from CF, with lower competitive abilities, remain in their natal, coniferous habitat patch. This size assortment is similar to analogous systems in which hole-nesting passerines born in marginal habitats that develop high-quality phenotypes are more likely to disperse into, and breed in, preferred nonnative habitats than individuals from marginal habitats with low-quality phenotypes (Verhulst et al. 1997; Braillet et al. 2002; Garant et al. 2005), with important consequences for phenotypic divergence (Bolnick et al. 2009). For instance, asymmetric exchange of birds from the low to the high-quality habitat underlies the differentiation in adult body mass and tarsus and wing length between two neighbor populations of Citril Finches (*Serinus citrinella*; Senar et al. 2002, 2006) whereas the differences in fledgling mass between nearby populations of Great Tits *Parus major* are due to larger immigrants being attracted to the habitat patches where densities are lowest (Garant et al. 2005). Although directional dispersal also caused phenotypic divergence in our study system, there has not been a gradual development in the asymmetry of male phenotypes (Garant et al. 2005). In contrast, the size assortment has been sustained along most of the study period (19 of 21 years) covered by our work, even when random movements of females in relation to phenotype should be contributing to swamp overall size differences between habitats. It is noteworthy, however, that phenotypic divergence, which may have evolved through natal dispersal, could be paradoxically maintained over time through quite the opposite process, as reassortment of individuals between habitats as a consequence of breeding dispersal is a very rare event (Fig. 4).

The size assortment of pied flycatchers seems to be mainly influenced by environmental factors, as shown by Shapiro et al. (2006) through partial cross-fostering experiments between high- and low-density populations of Great Tits revealing an absence of gene–environment interactions (Shapiro et al. 2006). Accordingly, the authors concluded that differences between parts of the population were due to phenotype-dependent dispersal over short distances. In our system, directionally biased dispersal may have contributed to increase overall breeding density in the deciduous plot, which might ultimately affect habitat quality and fitness (Kawecki and Holt 2002; Garant et al. 2005). In heterogeneous landscapes, optimal habitat features may not be evident at the time the choice of breeding habitat is made (Hutto 1985; Battin 2004), so preset preferences might explain why the larger, dominant males still prefer to abandon the pinewood patch over the increasingly overcrowded oakwood plot. In this way, individuals relegated to the coniferous forest might enjoy the advantages derived from reduced competition not accrued to males breeding in the ‘promising’ adjacent habitat (see e.g., Alatalo and Lundberg 1984; Gustafsson 1987; Mänd et al. 2005). This might lead to a perceptual trap scenario in the course of time (Mänd et al. 2005; Robertson and Hutto 2006).

To conclude, our results show how phenotypic differentiation between nearby subpopulations can be caused and sustained over relatively long time periods by nonrandom dispersal. The combined effects of the species' preferences in habitat choice and derived competitive interactions might give rise to differential demographic consequences. This highlights the role of behavioral and nongenetic, environmentally induced processes in driving evolutionary scenarios at small spatiotemporal scales, even when there are no apparent restrictions to gene flow.
